# Review: Sustainable Clinical Development of CAR-T Cells – Switching From Viral Transduction Towards CRISPR-Cas Gene Editing

**DOI:** 10.3389/fimmu.2022.865424

**Published:** 2022-06-17

**Authors:** Dimitrios L. Wagner, Ulrike Koehl, Markus Chmielewski, Christoph Scheid, Renata Stripecke

**Affiliations:** ^1^ Berlin Center for Advanced Therapies (BeCAT), Charité – Universitätsmedizin Berlin, corporate member of Freie Universität Berlin and Humboldt-Universität zu Berlin, Berlin, Germany; ^2^ BIH-Center for Regenerative Therapies (BCRT), Berlin Institute of Health (BIH) at Charité – Universitätsmedizin Berlin, Berlin, Germany; ^3^ Institute of Transfusion Medicine, Charité – Universitätsmedizin Berlin, corporate member of Freie Universität Berlin, Humboldt-Universität zu Berlin, Berlin, Germany; ^4^ Institute of Cellular Therapeutics, Hannover Medical School, Hannover, Germany; ^5^ Fraunhofer Institute for Cell Therapy and Immunology (IZI) as well as Institute of Clinical Immunology, University of Leipzig, Leipzig, Germany; ^6^ Clinic I for Internal Medicine, University Hospital Cologne, Cologne, Germany; ^7^ Laboratory of Regenerative Immune Therapies Applied, Research Center for Translational Regenerative Medicine (Rebirth), Department of Hematology, Hemostasis, Oncology and Stem Cell Transplantation, Hannover Medical School, Hannover, Germany; ^8^ German Centre for Infection Research (DZIF), Partner site Hannover, Hannover, Germany; ^9^ Cancer Research Center Cologne Essen (CCCE), Cologne, Germany

**Keywords:** CAR-T, lentiviral, retrovirus, gene editing, GMP, CRISPR-Cas, GMP, mouse models

## Abstract

T cells modified for expression of Chimeric Antigen Receptors (CARs) were the first gene-modified cell products approved for use in cancer immunotherapy. CAR-T cells engineered with gammaretroviral or lentiviral vectors (RVs/LVs) targeting B-cell lymphomas and leukemias have shown excellent clinical efficacy and no malignant transformation due to insertional mutagenesis to date. Large-scale production of RVs/LVs under good-manufacturing practices for CAR-T cell manufacturing has soared in recent years. However, manufacturing of RVs/LVs remains complex and costly, representing a logistical bottleneck for CAR-T cell production. Emerging gene-editing technologies are fostering a new paradigm in synthetic biology for the engineering and production of CAR-T cells. Firstly, the generation of the modular reagents utilized for gene editing with the CRISPR-Cas systems can be scaled-up with high precision under good manufacturing practices, are interchangeable and can be more sustainable in the long-run through the lower material costs. Secondly, gene editing exploits the precise insertion of CARs into defined genomic loci and allows combinatorial gene knock-ins and knock-outs with exciting and dynamic perspectives for T cell engineering to improve their therapeutic efficacy. Thirdly, allogeneic edited CAR-effector cells could eventually become available as “off-the-shelf” products. This review addresses important points to consider regarding the *status quo*, pending needs and perspectives for the forthright evolution from the viral towards gene editing developments for CAR-T cells.

## Introduction

Retroviruses integrate into the genome, are able to effectively and persistently infect T cells, and are non-cytotoxic and poorly immunogenic. Their bio-engineered descendants, pseudotyped gammaretroviral and lentiviral vector systems (RVs/LVs), emerged more than three decades ago as useful tools for gene therapies using T cells and hematopoietic stem progenitor cells (HSPCs) for correction of defective genes and treatment of monogenic blood disorders and metabolic diseases (1). RVs and LVs are currently the mostly used gene delivery systems for manufacturing of T cells expressing chimeric antigen receptors (CARs). Nonetheless, there were several ups-and-downs on the path to clinical translation of these “living drugs” that can instruct the development of gene-edited CAR-T cells generated by non-viral materials and the use of site-specific gene transfer.

**Figure 1:**
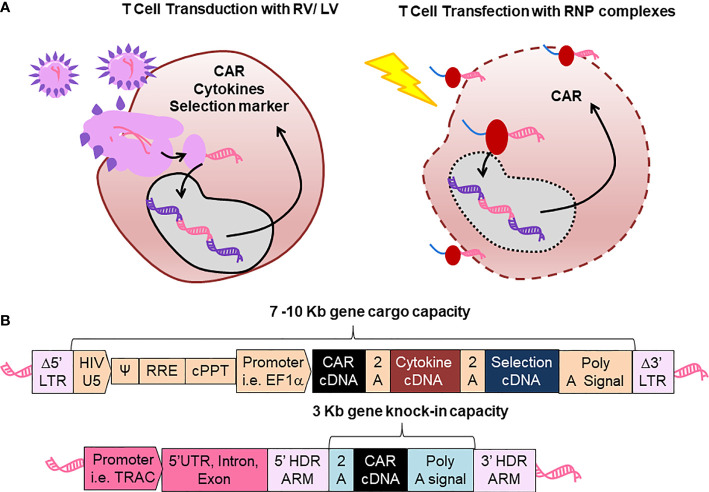
Comparison between retroviral vector and lentiviral vector (RV/LV) gene delivery systems with CRISPR-Cas gene editing for production of chimeric antigen receptor (CAR)-T cells. **(A)** Scheme of T cell transduction with RV/LV (left) and cell transfection with ribonucleoprotein (RNP, Right). **(B)** Schematic representation of genetic structures. Upper structure: Displays an integrated prototypic LV gene transfer vector encoding a CAR, not to scale. LTR: Long terminal repeats; HIV: Human immune deficient virus U5: Untranslated region in the 5’ side; Ψ: encapsidation signal; RRE, Rev responsive element; cPPT, polypurine tract; EF1α, Elongation factor 1 α. Lower structure: Represents a prototypic integrated CAR generated by gene editing. *TRAC*, Locus of T cell receptor alpha chain; HDR, Homology-directed recombination.

In 1990, the first clinical trial of gene-modified T cells used RV-mediated transfer of adenosine deaminase (ADA) for treatment of two children with severe combined immunodeficiency (ADA-SCID). The trial demonstrated engraftment, persistency and safety of the T cell gene therapy (2). Major improvements in efficacy and safety of multiple attenuated self-inactivating (SIN) RV/LV designs have significantly boosted the field of innate genetic defects corrected *via* gene therapy (3, 4). Thus, after more than two decades of clinical research and development, the European Commission granted market approval to GlaxoSmithKline (GSK) for *ex vivo* HSPC gene therapy for the treatment of ADA-SCID (5). The development of SIN viral designs drastically reduced the risks of insertional mutagenesis enabled better control of the transgene expression (6). These viral systems provided a robust insertion of a gene-of-interest (GOI), which was added to the genome of target cells (**Figure 1**).

Published clinical trial reports in 2011 and 2013 presented clinical objective responses against lymphoma and leukemia with CAR-T cells generated after SIN-LVs gene transfer (7, 8). To date, all CAR-T cell products approved by the United States Food and Drug Administration (FDA) and by the European Medicine Agency (EMA) for immunotherapy of lymphomas and/or leukemias are engineered *via* “add-on” transgenesis using SIN-LVs or SIN-RVs. These approved products target the B cell antigen CD19 including: LV-transduced CTL019 (KYMRIAH, Novartis Pharmaceuticals Corp) (8), RV-transduced KTE-C19 (YESCARTA, Kite Pharma, Inc., a Gilead Sciences Company) (9), RV-transduced brexucabtagene autoleucel (TECARTUS, Kite Pharma, Inc., a Gilead Sciences Company) (10) and LV-transduced liso-cell (BREYANZI, Juno Therapeutics, Inc., a Bristol-Myers Squibb Company) (11). LV-mediated gene delivery currently dominates CAR-T cell manufacturing. FDA/EMA-supported combination trials exploring alternative targets to CD19 (CD20, CD22, CD30, and the B cell maturation antigen, BCMA) are planned to improve efficacy in the CAR-eligible leukemia/lymphoma patient population (12). In addition, bi-specific CAR-T cells engineered with RV/LVs are in clinical testing (e.g. 2019-CD20-dual specific CAR-T cell product from Miltenyi Biomedicine) (13). In conclusion, SIN RVs/LVs have provided an important framework for the conception and clinical use of CAR-T cells as they are feasible and safe.

Alternative “add-on” transgenesis *via* DNA plasmid-based non-viral gene-modification technologies are being developed to replace viral systems in order to reduce the costs and facilitate the logistics of CAR-T cell manufacturing. CD19-specific CAR-T cells transfected with the Sleeping Beauty (SB) or *piggyBac* transposon showed exciting preclinical results (14) and promising results in early clinical trials (15). Sadly, unexpected and alarming insertional mutagenesis and T cell-lymphoma occurrences have been observed in some patients infused with CD19CAR-T cells produced with a highly active version of the *piggyBac* transposon system (16). Multiple transgene insertions and global transcriptional dysregulation through the strong promoters used are suspected to have caused the malignant transformation (15). Thus, additional preclinical studies and clinical monitoring efforts are urgently warranted for a better mechanistic understanding to prevent the onset and putative development of leukemias and lymphomas when using potentially pro-oncogenic transposon systems (17). Another pipeline in development is the transient transfection of T cells with mRNAs encoding CARs. Since the mRNA are degraded or diluted upon T cell expansion, the anti-tumor effect is predestined to be transient. The mRNA-CAR-T cell therapy would thus require repeated infusions, and it is yet not clear if this is a downside for this approach (18).

As an innovative alternative, clustered regularly interspaced short palindromic repeats (CRISPR) associated (Cas) 9 technology has emerged as a replacement for the “add-on” approaches with directed and precise T cell editing *via* “knock-in” (**Figure 1**). CRISPR-Cas allows the site-specific insertion of the CAR at potentially any point in the T cells genome, creating CAR-T cells with defined transgene copy numbers and predictable regulation of transgene expression. For example, the CAR transgene can be inserted within the early open reading frame of well-characterized genes, thereby disrupting the gene of interest (“knock-out”) after “knock-in” of the CAR in a single genetic intervention. This technology is exceptionally useful to facilitate potent “off-the-shelf” CAR-T cells to reduce costs and avoid treatment delays in severely compromised patients.

Under the headings below, we explain how RVs/LVs became the current paradigm for gene modifications of CAR-T cells. We address some of the critical aspects regarding the development of gene-edited CAR-T cells to thrive as a program for the treatment of liquid and solid tumors. One important focus is on what was learned about the design, safety, manufacturing, upscaling, and quality control of CAR-T cell products generated with RV/LVs and the perspective for gene edited CAR-T cell products. In a next step, we extrapolate towards the need for new preclinical models, innovative design of clinical trials and monitoring of patients infused with allogeneic “off-the-shelf” gene-edited CAR-T cells.

## Principles and Uses of RVs/LVs for CAR-T Cell Engineering

Bioengineering of RV/LV systems for gene-modification of HSPC products has provided the fundamental know-how for subsequent development of CAR-T cells. RVs/LVs have relied mostly on the third generation packaging system, whereby four plasmids are used for expression of the backbone vector, *gag*/*pol*, *rev* and *env* (19, 20). After infection of the activated and proliferating cells, the RNA genomes of RVs/LVs are converted through reverse transcription inside the cell into double-stranded DNAs capable of integrating into the chromatin (**Figure 1A**). Thus, the core of multicistronic RV/LV engineering is that a single vector will carry the combination of genes into the cells, however with quite unpredictable insertion patterns. An improvement was obtained with SIN mutations in the viral 3’LTR, disrupting the promoter/enhancer activity of the LTRs and enhancing the controlled expression through the internal promoter in the vector, and thereby minimizing the downstream expression of proto-oncogenes that could promote insertional mutagenesis (3). The design of RVs/LVs mostly include viral elements needed for packaging (parts of the LTRs, Ψ psi encapsidation signal), RNA reverse transcription (central polypurine tract, cPPT), internal non-methylated promoters (e.g. EF1-α), and the GOI (21) (**Figure 1B**). Since the gene cargo capacity of RVs/LVs spans from 7 to 10 Kb, additionally to the CAR (around 3 Kb), other transgenes can be combined as multicistrons interspaced with a 2A self-cleaving peptide or with internal ribosome entry site (IRES) elements.

As a result, there are numerous synthetic biology strategies relying on RV/LV systems to optimize the CAR gene-cargo, which include (i) tuning the affinity of the virally expressed CAR(s) to antigen(s) (22), (ii) use of different intracellular co-stimulatory domains in the CAR fusion protein such as CD28z, 4-1BB and other co-stimulation pathways directing the tonic power and/or persistency of T cell activation (23); (iii) metabolic editing to balance the oxidative phosphorylation and fatty acid oxidation or glycolysis during T cell activation (24); (iv) combinatorial co-expression of immune-stimulatory cytokines to improve the T cell persistency and function (25); and (v) inclusion of inducible on/off systems such as co-expression of suicide genes, surface markers that enable immune depletion, or combination of activation/inhibitory CARs in the same cell (22).

The clinical performance obtained for RV/LV-engineered CAR-T cells in the treatment of B cell malignancies has not yet been achieved in the treatment of solid tumors. The main difficulties encountered are the lack of exclusive tumor-specific antigens and the immunosuppressive nature of the tumor microenvironment (26). Although the challenges may rely rather on tumor-specific factors than the technology used for CAR gene-delivery, gene-editing may replace RVs/LVs for different approaches. For example, sophisticated tumor detection and targeting advances can be achieved by engineering T cells with CAR constructs expressed by RVs/LVs to function as comparative operators (27–29). Promising approaches based on the so-called TRUCK (“T cells redirected for antigen-unrestricted cytokine-initiated killing”) strategy have recently emerged to increase the efficacy of CAR-T cells generated after RV transduction (30–32). TRUCKs combine the direct cytotoxic effect of the CAR-T cell on tumor cells with the immune modulating capacities of a pro-inflammatory cytokine (33). In order to achieve therapeutic concentrations of a selected cytokine in tumors and surrounding tissue, the transgene of interest is inducibly released by tumor-specific CAR-T cells, thereby preventing systemic toxicity. The TRUCK concept is currently being explored using a panel of pro-inflammatory cytokines, including interleukin (IL)-12, IL-15, IL-18, IL-23, and combinations thereof (33).

## Large-Scale Manufacturing of Clinical-Grade RVs/LVs

The large-scale manufacturing of RVs was initially based on development of stable packaging cell lines. During the past two decades, with the advent of the third generation LV packaging systems, the field has largely explored transient transfection of different DNA plasmids into packaging cells (such as adherent or non-adherent HEK293T cells). Transient transfection for packaging of RVs/LVs became an important technology as it bypasses the need of selecting, expanding and characterizing different master packaging cell lines carrying different constructs. RVs/LVs obtained after transient transfection were validated for different types of clinical applications such as gene modification of HSPCs for correction of defective genes (1), for harnessing dendritic cells for active immunotherapy against cancer (34, 35) and, more prominently, for gene modification of T cells for different types of adoptive immunotherapies (21).

The large-scale bioprocessing of LVs has in recent years adopted the use of bioreactors for perfusion transfection and culture of adherent and suspension cells. Several advances were obtained with the downstream processing of the viral particles with purification technologies (such as tangential flow filtration) (36, 37). Quality control (QC) of SIN-LVs is well established and includes: Vector identity (qPCR), Vector concentration/titer (ELISA), Vector functional titer (flow cytometry), residual plasmid DNA (VSV-G DNA qPCR), Residual host DNA (antigen-specific qPCR), detection of replication competent lentivirus (RCL), quantification of residual Benzonase (ELISA), total protein measurements (protein assay), microbiological control (bacteria and fungi assay), detection of endotoxin (LAL assay) as well as volume, pH and appearance (36).

Several obstacles still limit the applicability of large scale use of clinical LVs for medical care. The high costs of LVs for production of T cell therapies, is an important bottle-neck contributing to exorbitant costs of the cell products for a single treatment course (currently >300.000 US dollars in the United States) (37). Further, due to the currently limited manufacturing capacity for LVs, the commercially available CAR-T cell therapies are only regarded as a second-, third- or fourth-line therapeutic option for patients failing to respond to, or have relapsed after conventional therapies (37).

## Clinical Manufacturing of CAR-T Cells Generated With LVs/RVs

A major challenge for academic institutions, such that CAR-T cells become a standard clinical strategy, is to scale out the GMP-compliant manufacturing (38–41). The entire manufacturing process for semi-automated or automated processes requires 12 days (range 6-22 days) (39, 42). The subsequent procedures include T cell activation, gene transduction, expansion and often cryopreservation after the final formulation. First, T cells, selected (e.g. CD4^+^ and CD8^+^) or not, are commonly activated with agonistic anti-CD3 and anti-CD28 antibodies and expanded in the presence of stimulatory cytokines (mostly IL-7, IL-15 and/or IL-2) for 1-2 days (38). Afterwards, the viral vectors are added to the cell culture system, often in the presence of cationic adjuvants to enhance the transduction efficacy. Prior to large-scale CAR-T cell manufacturing, pilot lots are tested with different vector dosages to yield a satisfactory multiplicity of infection (M.O.I.), i.e., resulting in 5 or less viral copies per cell. After transduction, CAR-T cells are expanded in culture with cytokines for additional 5-10 days. Optimized GMP protocols using RVs/LVs have resulted in high gene delivery efficacy, resulting a range of 25-80% CAR-positive T cells including both CD4^+^CD3^+^ and CD8^+^CD3^+^ CAR-T cells after transduction and expansion. Since LV gene transfer is usually robust in actively replicating T cells, manual manufacturing methods can be efficiently replaced with closed automated systems (42, 43). Importantly, digitally controlled automated manufacturing systems can potentially improve the practicability and lower the costs associated with clean rooms and highly trained personnel for production of CAR-T cells for a broader patient usage (41). Thus, in sum, although the upstream production and testing of clinical grade RVs/LVs still remains complex and expensive, the downstream T cell transduction procedures are relatively straightforward, particularly with the launching of powerful automated cell manufacturing systems allowing consistent gene transfer efficacy, cell recovery and viability (**Table 1**).

**Table 1:** Comparison of technical ease, elements needed, procedures, and efficacies between retroviral vector and lentiviral vector (RV/LV) gene delivery systems with CRISPR-Cas gene editing for production of chimeric antigen receptor (CAR)-T cells.

	RV/LV	CRISPR-Cas RNP
Generation of Gene transfer system	Viral packaging and purification, customized,complex, costly	Highly adaptable and modular, RNA/ DNA synthesis and recombinant protein, simple
QC of gene transfer system	Complex molecular biology and virology, biochemical, biological tests	Simple biochemical synthesis and biochemical tests
PBMC/T cell activation	1-2 days	1-3 days
T cell modification	Virus plus adjuvant, overnight incubation	Several reagents, electroporation and resting
T cell expansion	>1000 fold relative to input	Up to 200 fold relative to input
Insertion in genome	Mostly random and in pro­ oncogenic hotspots	Targeted to specific loci but off­ sites possible
Multicistronic gene transfer	Feasible within gene cargo capacity	Remains to be optimized
Production of HLA-KO Allogenic CAR-T cells	Feasible with shRNAs or gRNAs expressed in viral vector, and with electroporation of mRNAs expressing TALENs	Feasible with gRNAs included in gene editing procedure

## Transgene “Knock-In” With CRISPR-Cas Gene Editing

The 2020 Nobel Prize for Chemistry was awarded to Jennifer Doudna and Emmanuelle Charpentier, eight years after their original publication describing how the CRISPR RNAs (crRNAs) can guide recombinant Cas9 enzymes to recognize, bind and cut a DNA sequence of interest *in vitro* (44). They elucidated how a mature crRNA base-paired to trans-activating crRNA (tracrRNA) was able to form a duplex RNA structure, which guides the CRISPR-associated *Streptococcus thermophilus* and *Streptococcus pyogenes* (Sp)Cas9 proteins to the target DNA where it then introduces double-stranded (ds) breaks. They also demonstrated that dual-tracrRNA:crRNA when engineered as a single RNA chimera could also direct sequence-specific Cas9 dsDNA cleavage (44). The high flexibility and efficacy of the RNA-guided nuclease CRISPR represents a disruptive technology which has opened several doors for synthetic biology and cell therapies.

The use of a programmable nuclease to precisely edit DNA at specific loci was then used by Eyquem et al. to replace the endogenous T cell receptor (TCR) alpha chain with a CAR. They combined transfection of anti-CD3/CD28-stimulated T cells with Cas9-single guide (sg)RNA ribonucleoprotein (RNP) complexes followed by transduction with a recombinant adeno-associated virus serotype 6 (rAAV6) to deliver the DNA donor template and homology-directed DNA repair (HDR) arms for CAR integration into the first exon of TCR-α constant gene (*TRAC*) (45). They observed homogeneous CAR expression in human T cells and *TRAC*-integrated CAR-T cells therapeutically outperformed CAR-T cells generated *via* RV transduction in a preclinical mouse model of acute lymphoblastic leukemia. Improving the design of the CD19-CAR was shown to further increase the potency of *TRAC-*replaced CAR-T cells in leukemia and lymphoma models (46). Subsequently, these advances were adopted by other groups for use of CAR-T cells in the context of haploidentical stem cell transplantation (47).

Fully non-viral gene editing approaches with DNA templates for CAR/TCR knock-ins are rapidly emerging (48) (**Table 2**). Roth et al. demonstrated the use of virus-free knock-in to replace the endogenous TCR with an ectopic TCR targeting the NY-ESO-1 cancer antigen (54). Cas9 RNPs were co-electroporated with a blunt-ended dsDNA HDR template (HDRT) with homology arms designed to introduce the α and β chains of the TCR into the *TRAC* gene (54). The resulting TCR-engineered T cells specifically recognized NY-ESO-1 and killed tumor cells expressing NY-ESO-1 *in vitro* and *in vivo*. Interestingly, the gene edited T cells engineered with the CRISPR-Cas system mounted better antitumor immune responses in a mouse model than T cells gene modified with lentiviral vector expressing the same TCR, probably because they could be better self-regulated to avoid exhaustion.

**Table 2:** Examples of prominent studies using CRISPR system for genetic modification of T cells to produce CAR-T cells.

Reference	Target Antigen and co-stimulation	Target Genetic Locus	Methods for Gene Editing	Frequency of CAR+ T Cells after Knock-in	Potency Assays *in vitro* and *in vivo*
Eyquem et al. Nature 2017 (45)	CD19 CD28 zeta	*TRAC*	sgRNA and Cas9 mRNA AAV-mediated HDR	Up to 40%(10e6 AAV dose)	*In vitro* culture with Nalm-6/fLuc/GFP or NIH-3T3/CD19
		*B2M*	sgRNA-Cas9 mRNA AAV-mediated HDR	14%	*In vivo* Nalm-6/fluc/GFP xenogra fted in NSG male mice
Feucht et al, Nature Medicine 2019 (46)	CD19 CD28 Zeta (+ITAM-mutated versions)	*TRAC*	sgRNA and Cas9 mRNA AAV-mediated HDR	60-75%	*In vitro* culture with Nalm-6/fLuc/GFP or NIH/3T3/CD19 *In vivo* Nalm-6/fLuc/GFP xenograftedin NSG male mice
Wiebking et al, Haematologica 2021 (47)	CD19 CD28 zeta	*TRAC*	sgRNA-Cas9RNP AAV-mediated HDR	>70%	*In vitro* co-culture cytotoxicity assays & cytokine production from supernatants (ELISA) *In vivo* Nalm-6/fLuc/GFP xenograft in NSG mice
Roth et al. Cell 2020 (49)	Different chimeric receptors (pool) + TCR e.g. TGFβR2-41BB	*TRAC*	SgRNA-Cas9 RNP dsDNA-mediated HDR	5-6%	*In vitro* expansion, co-culture killing assay and *in vivo* solid tumor A375 melanoma xenograft in NSG mice
Ode et al. Cancers 2020 (50)	IL13Rα2 CD28	*TRAC*	sgRNA-Cas9RNP dsDNA-mediated HDR	20% (but low expression level)	none
Kath et al, Biorxiv preprint 2021 (51) *In press Mol Ther Meth Clin Dev 2022*	CD19 CD28 zeta	*TRAC* *AAVS1*	sgRNA-Cas9RNP dsDNA-medai ted HDR sgRNA-Cas9RNP dsDNA-medai ted HDR	25-68%(enhanced by drug co- treatments) 10-15%	*In vitro* co-culture cytotoxicity assays & intracellular staining of effector cytokineproduction *In vivo* Nalm-6/fLuc/GFP xenograft in NRG mice
Muller et al. Frontiersin Immunology 2021 (52)	HLA-A2 CD28 zeta	*TRAC*	sgRNA-Cas9RNP dsDNA-mediated HDR	ca. 8-10% (increased during expansion up to 90%)	*In vitro* assays for Treg function (phenotyping, activation status, proliferation suppression) *In vivo* mouse model of GvHD and xenogeneic GvHD
Jing et al. Small Methods 2021 (53)	CD19 or CD19/CD22 CD28 mutZeta or Zeta	*TRAC*	sgRNA-Cas9 RNP Minicircle pDNA-mediated HDR sgRNA-Cas9 RNP AAV-mediated HDR	10-18% (with two Cas9-target sequences in donor template & recombinant Cyclin D protein) No details regarding Kl rates	*In vitro* expansion, co-culture cytotoxicity assays. (Nalm-6/fLuc/GFP) *In vivo* Nalm-6/fLuc/GFP xenograft in NSG mice

Both the automated and large-scale chemical production of the gRNAs and novel enzymatic techniques to synthesize the HDRT have sky-rocketed in recent years. Although still costly at the clinical stage, a large set of CRISPR products are broadly available for basic research from multiple commercial vendors. The number of manufacturers that provide GMP services for Cas enzymes and customized gRNAs or DNA templates is starting to expand, and due to demand and competition, will likely become more affordable for clinical use in the years to come. Since these products are chemically defined, the quality control analyses will be mostly chemical/biochemical. Furthermore, CRISPR-Cas related reagents have extraordinary stability. Some studies have successfully lyophilized defined RNP/DNA composition, which could further improve roll-out of the technology (55).

Unlike RVs/LVs, the RNP complexes used for gene editing lack the machinery to cross the cellular membrane and reach the chromatin within the intra-nuclear space (**Table 1**). Most published protocols use electroporation as means to introduce the RNP into the cell with minimal toxicity to T cells (56, 57) (58). However, co-delivery of large dsDNA donor templates required for CAR knock-in induces significant dose-dependent toxicity (50, 54) (**Table 2**). Physical shear stress, DNA damage responses as well as innate immune responses to free cytosolic RNA or DNA, endanger cell viability, gene modification and ultimately a good recovery of CAR^+^ viable T cells. In contrast to dsDNA, TCR-knock-in with ssDNA donor templates is less toxic, however with significantly reduced integration rates compared to dsDNA for pooled CAR knock-ins (49). Use of anionic adjuvants that disperse RNPs have been shown to reduce toxicity and increase efficacy of virus-free reprogramming with large dsDNA donor templates (55).

As a result of different optimization steps, in most publications on virus-free TCR/CAR knock-ins blunt-ended dsDNA or plasmids were used with the frequency of knock-in T cells reported in a range between 5-50% after 7-14 days of expansion (49, 50, 51, 52, 53). Based on experience of authors of this review, the number of recovered T cells 10 days after initiation of the editing procedure can reach 10-200 times the number of PBMCs used as input (51). Initial cell loss after electroporation and the modest expansion rate observed remain limiting factors warranting innovative technologies. These could include nanocarriers, liposome or virus-like particle-based delivery platforms for DNA and/or RNPs which circumvent electroporation procedures. Furthermore, synthetic DNA donor templates may be optimized or enhanced to decrease toxicity, increase efficacy, and reduce the risk for unintentional integration events.

In conclusion, the materials used for virus-free CRISPR-Cas editing are and will be easier to produce, store and distribute for clinical use than large-scale manufacturing of RVs/LVs. The current challenge is to further optimize and standardize the gene editing procedures to improve CAR T cell yields and manufacturing stability. Subsequently, virus-free knock-in methods should be adopted for automated manufacturing systems to accommodate future clinical scaling (41, 59).

## Clinical Quality Control and *in Vitro* Potency Analyses of CAR-T Cells

In process and end process QC of CAR-T cells gene-modified with RVs/LVs include tests for cell identification (T cell number, cell viability, phenotypic characterization, expression of CAR or other transgenes), impurity measurements and safety (sterility, mycoplasma, endotoxin). More comprehensively, fluorescent-activated cell sorting (FACS) analyses of cell count, cell composition and transduction rate are established using basic panels including staining for CD3/CD4/CD8/CD14/CD16 CD45/CD56. A viability dye, such as 7AAD, is used for exclusion of dead cells. The panel also includes antibodies binding to the extracellular domains of the CAR-specific detection antigens (i.e., binding to the single-chain fragment variable, or scFv) in order to quantify the CAR expression levels and to determine the frequency of CAR^+^ cells within the T cell subpopulations (42). In addition, transduction efficiency can also be determined by quantitative PCR. Although highly unlikely due to the use of SIN vectors, testing for the presence of replication-competent RV/LV particles (replication-competent retrovirus (RCRs) or the counterpart RCLs) by quantitative polymerase chain reaction (qPCR) is mandatory. Besides the above described parameters, the DNA encoding the VSV-G viral envelope (that can be carried by the transduced cells) is quantified using real time qPCR (according to the European Pharmacopeia). In addition, *in vitro* potency assays are needed, such as co-culture of CAR-T cells and target cells and measurement of IFN-γ and other cytokines into the medium supernatant in response to T cell activation.

In addition to these validated batch-release QC parameters, several other optional analyses can be included as monitoring only for research purposes. In this respect, FACS-based multiparametric immunophenotyping is used in order to characterize cell subpopulations including fitness of the cells, naïve/effector and central memory T cells as well as expression of co-stimulatory and inhibitory checkpoint receptors. Quantification is done in both the final CAR-T cell product and in the peripheral blood of patients for immune monitoring of CAR-T cell persistence (60).

In contrast to the QC analyses of CAR-T cells generated *via* RV/LV transduction as described above, there is currently little clinical experience with CRISPR-Cas gene edited T cell products (61–64). Overall, gene-edited CAR-T cell products will require the same validated batch-release QC parameters as RV/LV-transduced CAR-T cell products. Clinical release criteria of CAR-T cells engineered by knock-in into the *TRAC* locus should be complemented by a stringent FACS assessment of residual T cells expressing TCR-α/β^+^ in the final product. Additionally, quantification of residual xenogeneic Cas9 protein may be performed to avoid immunogenicity risks during short-expansion protocols (65). In our experience, Cas9 is usually rapidly diluted and degraded after transfection in highly proliferating T cells within just a few days (66).

Preclinical assessments and monitoring for research differ dramatically for gene-edited CAR T cells: The current main safety concern of gene-edited CAR T cells is related to unintended consequences of the nuclease activity, including off-target editing and chromosomal aberrations such as large deletions or translocations. Therefore, preclinical QC must include careful selection and off-target profiling of the gRNA and respective Cas enzyme. Regulators commonly request a set of assays to identify potential off-targets in the genome, which can include *in silico* prediction with computational tools, but must also include unbiased experimental approaches (67), which have been reviewed extensively elsewhere (68). Subsequently, in depth analysis of putative off-target sites must be performed typically by next generation sequencing (NGS). Large on-target deletions as well as other chromosomal arrangements are usually not detected by amplicon-based sequencing of predicted off-targets (69, 70). As standard karyotyping may not have the necessary sensitivity to identify these aberrations, novel NGS-based approaches including CAST-seq, a sensitive assay for identification and quantification of unintended chromosomal rearrangements have been developed (71). Clonality analysis at the preclinical stage may inform on excessive outgrowth of cell clones harboring driver mutations. However, recent evidence from a clinical trial with multiplex-gene edited T cells reported that cells harboring translocations between the intended cut-sites were lost, indicating decreased cell fitness of the aberrant cells (61, 62). Of note, as random integrations of double-stranded DNA templates are rare, HDR-based gene insertion has significantly reduced risk for insertional mutagenesis over RV/LV (49). Past experience with *in vitro* assays for prediction of insertional genotoxicity was established for RV/LV systems and this knowledge can be applied to formally prove the safety of gene editing (72).

Exploiting endogenous transcription programs by gene editing knock-in can further circumvent the need for viral promoters or promoters that lead to supra-physiologic transcriptional activity and that can impact the expression of neighboring genes (73). Therefore, in order to predict and assess long-term safety of gene-edited CAR-T cells, forward-looking and validated assays that allow quantification of off-targets or translocations will be highly important. *In vitro* potency assays for gene-edited CAR-T cells can be largely adopted from previous experience listed above for CAR-T cells generated with RVs/LVs. Remarkably, analysis of cytokines released or cytotoxicity effects after co-culture of CAR-T cells generated by knock-in into *TRAC* with target cells may show increased antigen-specific reactivity, most likely because the TCR^neg^ CAR-T cell product lacks allo-reactive effects. This is an important finding, as TCR^neg^ CAR-T cells can be tested against panels of several patient-derived primary tumor cells. Allogeneic CAR-T cells could be recognized by the recipient patient’s immune system which can limit their therapeutic efficacy by preventing cell persistence or reducing effector functions (74). Certain patient populations, including transplant recipients or heavily transfused patients, may already have allo-specific antibodies, which could inactivate off-the-shelf CAR-T cells. Careful matching of healthy-donor or additional genetic interventions may circumvent this problem. Standard assays to evaluate allogeneic cell compatibility including screening of patient serum for presence of antibodies recognizing the major histocompatibility complex (MHC) or other features of the allogeneic CAR-T cell product could be included to select a suitable gene-edited CAR-T cell product based on the patients given allo-sensitization (74).

## Preclinical Models for Testing CAR-T Cells and Off-the-Shelf CAR-T Cells

The *in vivo* response to CAR-T based immunotherapies varies due to substantial molecular heterogeneity and immune suppressive pathways of human tumors and the poorly understood mechanisms that determine CAR-T efficacy as well as to predict side effects (75). Nonetheless, preclinical mouse models used to demonstrate efficacy of CAR-T cells generated after RV/LV transduction were indispensable for their subsequent evaluation in clinical trials and ultimately for their clinical approval.

In general, the first proof-of-concept models use cell-line derived xenograft (CDX) tumor models. Cell lines are commercially available from repositories for comparative studies performed by different laboratories and some molecular pathways associated with cancer in the cell lines are well defined. For example, studies by Brentjens, Sadelain et al, second-generation CD19CAR-T cells (with the CD28zeta costimulatory domain) produced after RV transduction were validated *in vivo*, in SCID-Beige mice implanted intravenously with Nalm-6 cells expressing firefly luciferase (fLuc). The injected cells that develop into B-cell acute lymphoblastic leukemia (ALL) in mice and can be monitored by optical imaging analyses (76). In the Nalm-6 model, ALL disease is systemic with involvement of the bone marrow and central nervous system (76). Studies by Tsukahara et al. evaluated the accumulation of CD19-CAR RV-modified T-cells in Burkitt’s lymphoma lesions that develop in lymph node structures after i.v. implantation with the cell line Raji/fLuc (77). The Nalm-6/fLuc and Raji/fLuc xenograft models are useful models that are still commonly used for comparative evaluation of new designs of CD19 CAR-T cells targeting leukemias and lymphomas generated after viral gene delivery or by gene editing (54, 78).

However, immortalized cancer cell lines, either expanded *in vitro* or grown as xenograft tumor models, cannot reflect the real complexity of human tumors and only provide limited insights into human malignancies (79). The cell lines do not accurately reflect the primary tumor in gene expression and tissue composition as they have been cultivated in laboratories for many years or even decades (80). Therefore, preclinical studies on such lines are not sufficient to offer personalized and well-differentiated CAR-T cell immunotherapy in the future. As a dynamically emerging field, collections of primary tumors grafted into immunodeficient mice, patient-derived xenograft (PDX) mouse models. The mouse strain used for PDX-based studies is a very important determinant for the engraftment of cells for development of xenograft tumors. Several xenograft models are currently exploring non-obese diabetic (NOD)-scid mice or their derivatives because fewer human cells are phagocytosed by mouse macrophages (81). Further, a mutation in the interleukin 2 (IL-2) receptor common gamma chain *(Il2rγ)*, resulted in the NOD-*scid-IL2rγ^(-/-)^* (NSG) mouse strain lacking murine T and B cells and as well as natural killer (NK) cells (82). Thus, effective engraftment of different tumor cell lines in the NSG and in the related NOD/Shi-*scid IL2rγ^(-/-)^* (NOG) mouse strains has been adopted in several laboratories for evaluation of CAR-T cells produced after RV/LV transduction (79). Milone, June et al. initiated the innovative use of NSG mice implanted with primary ALL cells to test CD19CAR-T cells with the CD28 and/or 4-1BB intracellular domains generated by LV transduction (83). CD19CAR-T cells containing 4-1BB-ζ showed higher anti-leukemic efficacy compared to CD19CARs containing CD28-ζ signaling receptors and were significantly more persistent *in vivo* (83). Such mouse models using primary tumor samples reveal a more differentiated view on inter- and intra-tumor heterogeneity and more closely resemble the patient’s tumor in terms of histopathologic and molecular properties, as well as response to selected therapy. In particular, solid tumor types such as lung cancer (80), breast cancer (84) and gastric cancer (85) associated with vascular, mesenchymal and inflammatory architecture can be better recapitulated *in vivo* with PDX-based xenograft models. These preclinical models reflecting tumor heterogeneity are key for obtaining an understanding of how this heterogeneity affects responses to CAR-T cell immunotherapy and how it may change during treatment both at the genomic and at the phenotypic levels (86–90).

However, although the abovementioned models are extremely useful, they have a major limitation. CDX and PDX models are primarily generated in immunodeficient mice. The absence of essential elements of the human immune system in these mice limits the significance of such models to investigate the role of the immune system and interactions with CAR-T cells in anti-tumor responses, safety and immune toxicity. Immunodeficient mice transplanted with human hematopoietic stem cells (HSCs) are considered “fully humanized” human immune system (HIS) models since, after several months, they reconstitute a humanized immune system. Human HSCs engraft in the bone marrow and then differentiate systemically into several types of human hematopoietic lineages, such as mature leukocytes and myeloid cells, despite the full mismatch between the human leukocyte antigens (HLAs) expressed on the human hematopoietic cells and the mouse MHC expressed on tissues. Humanized mice are new animal models designed to address some of these efficacy and safety risks associated with cytokine release syndrome, thereby making them an attractive alternative for preclinical immunotherapy research (79, 91).

Allogeneic gene-edited TCR^neg^ HLA-I^neg^ HLA-II^neg^ CAR-T cells I will require preclinical efficacy testing in mice expressing HLAs matched to the tumors. Further, since cells lacking expression of HLAs can be targeted by natural killer (NK) cells, humanized mouse models with NK cells and that simulate the tumor microenvironment will substantially facilitate basic and translational research on allogeneic gene-edited CAR-T cell-based immunotherapy (92, 93).

## Design of Clinical Trials for Testing Allogeneic Gene-Edited CAR-T Cells

To date, several thousand patients have been treated or included in trials testing autologous RV/LV transduced CAR-T cells (94). Although allogeneic gene edited CAR-T cells may ease the procurement of CAR-T cells for patients in urgent need, the clinical trials will have to address several new aspects. For CAR-T cells produced after RV/LV transduction, the efficacy of the T cell therapy is associated with parameters such as disease indication, numbers of CAR-T cell product administered per kilogram (95). However, if the efficacy of the allogenic gene-edited CAR-T cells is substantially higher or lower, these associations would need to be re-evaluated. The major advantage of the allogeneic CAR-T cells for clinical study designs is that the product of one donor can be tested simultaneously in different subjects, which may result in more consistent data per donor-derived product. However, there may also be significant batch-to-batch product differences due to donor characteristics. Of note, one study could demonstrate that healthy donor-derived CD19CAR-T cells outperformed autologous leukemia patient-derived CAR-T cells in an *in vivo* xenograft model (96). Multiple reasons could explain the phenomenon: i) damage introduced by prior chemotherapy regimen, because patients were refractory to standard of care; ii) patient-intrinsic defects in effector immunity, which contributed to cancer development in the first place.

Importantly, clinical trials with autologous CAR-T cells produced after RV/LV transduction have established a clear toxicity profile, in particular cytokine release syndrome (CRS) and immune-effector cell associated neurotoxicity (ICANS) (97). With optimized clinical management standards, the rates of severe CRS and ICANS were markedly reduced and the results have been crucial to further expand the extended clinical application of CAR-T cells, e.g. in an outpatient setting. Of note, the timing of these complications can vary substantially between different CAR-T products, even for the same target. For BCMA targeted CAR-T therapies, CRS occurred within 1-7 days after infusion (98, 99). Similarly, for CD19 CAR-T cell therapies, the rate of neurological complications showed striking differences between two different cell products (100, 101). Thus, allogeneic gene-edited CAR-T cells will need to be benchmarked against these clinical results for CRS and ICANS obtained with autologous CAR-T cells, particularly because new immune-toxicities may emerge.

Regarding geno-toxicity, CAR-T cells generated with RV/LV transduction have shown an excellent safety profile. However, a recently, a trial of HLA-matched allogeneic CAR-T cells generated with the *hyperPiggyBac* transposon system, two out of ten children developed CAR-T derived lymphomas (102). Detailed genetic investigations were performed on biopsy material from tumor cells to elucidate the underlying pathogenesis. A high frequencies of genomic integration sites were found (16). Notably, in both lymphoma cases, *BACH2*, a gene involved in regulation of T cell plasticity, was downregulated with integration sites found within the *BACH2* locus (102). Although the mechanism of gene delivery by *hyperPiggyBac* and non-viral gene editing are different, these occurrences provide a note of caution regarding genotoxicity, as some loci may be hot-spots for insertional mutagenesis *via* HDR mechanisms.

Graft-versus-host-disease (GvHD) is not an issue in autologous CAR-T trials, however, if TCRs remain intact in allogeneic CAR-T cells, GvHD could become an additional relevant toxicity. In this case, it may have a different clinical presentation compared to GvHD presentation after allogeneic stem cell transplantation. Biopsies in affected tissues could inform about relevant cellular infiltrates. Further, lymphodepletion regimens may have to be optimized to enable a high engraftment of allogeneic CAR-T cells.

In addition to response rate and progression-free survival as typical efficacy endpoints, CAR-T cell persistence and clonality are important parameters to assess in clinical trials. This is typically done by assessing the CAR on T cells by flow cytometry or PCR amplification of the corresponding gene insertion in peripheral blood mononuclear cells. In contrast to the early expansion phase, quantification may be hampered at later stages because of the detection limit of these assays, in particular when CAR-T cells migrate to tissue niches. There is much greater genetic diversity between host cells and gene-edited allogeneic CAR-T cells which may hamper their persistence, but this could also be exploited for detection purposes. In addition to analysis of the CAR, analysis of HLA chimerism could be performed. Although MHC mismatches can be potentially eliminated by the knock-out of HLA class I and II, minor histocompatibility complexes and other polymorphic proteins can still potentially lead to allo-sensitization and rejection of the gene–edited CAR-T cells (74).

While these new complexities and additional safety risks of allogeneic CAR-T cells must be acknowledged, there are also significant advantages: Allogeneic CAR-T cells may be produced in large batches from healthy donor apheresis products and be made available as “off-the-shelf” products. This will dramatically shorten the delay between the decision to initiate CAR-T therapy and the actual delivery of the treatment. Currently, it may take up to 3 months from obtaining a production slot, organizing the apheresis, and shipping to the cell manufacturing facility, receiving the product, and infusing into the patient. Allogenic CAR-T cells may be available within a few days or even hours if stored at the site of care. In addition, the production of several batches from a single apheresis may substantially lower the cost of this treatment modality and thus alleviate the financial burden of CAR-T therapy.

## Paving the Way for Gene Edited CAR-T Cells: Outlook and Concluding Remarks

When considering a switch towards more innovative gene delivery approaches, i.e. from RV/LV systems to gene-edited non-viral CAR-T cells, several challenges need to be addressed until their broad clinical application:

- CAR-T cell performance will depend on the nature and location of transgene insertion. Identification of the optimal locus to allow for reasonable CAR expression level and its physiological regulation is paramount. As cargo payloads for HDR at a single locus are limited to the DNA repair mode (i.e., HDR), compact CAR formats and multicistronic knock-ins may be a first step toward enhanced CAR-T cells. However, improving the respective genetic cargo capacity using novel gene editors (e.g. CRISPR-integrases) or enhancing our ability for multiple knock-ins in a single CAR-T cell product will be needed for certain indications (e.g. solid cancers).
- The quality and safety of gene-edited/knocked-in CAR-T cells will largely depend on the gene editors used and what loci are targeted. Careful designs of gRNAs and HDRTs must be performed to avoid off-target effects and prevent insertional mutagenesis.
- The feasibility for clinical use is presently still limited by the relatively low number of recovered gene edited CAR-T cells as discussed above. New manufacturing and downstream technologies are required to decrease toxicity during gene editing. These could include, improved physical transfection systems, novel chemical transfection agents (e.g. lipid-nanoparticles) or pharmacological strategies to lower the cytotoxic effects of DNA double strand breaks that occur in the editing process. Furthermore, automated cell production, efficient expansion of T cells with favorable differentiation state and viable cell banking (for off-the-shelf purposes) are needed for success at clinical stage.
- Ultimately, the clinical potency of gene edited CAR-T cells will be strongly correlated with their *in vivo* activation upon antigen detection and persistence for long-term antitumor surveillance. Herein, deducting the optimal strategy to improve allogeneic CAR-T cell persistence in immunocompetent hosts will be key to success: Choosing the right tool for genetic engineering, establishing advanced host conditioning protocols and potentially adding HLA-matching procedures are possible ways forward. The prospect of future off-the-shelf products will also require solid logistics for manufacturing, cryopreservation and distribution.
- The development of predictive *in vitro* assays and humanized mouse systems must be further enforced by the community to benchmark antitumor efficacy and safety (e.g. CRS, GvHD) of novel gene-edited CAR-T cell candidates. Due to the abundance of potential strategies to enhance gene-edited CAR-T cells in the future, stable and reproducible models are paramount to prioritize them in the translational efforts and early clinical trials.

Following the philosopher George Santayna’s wise words “those who cannot remember the past are condemned to repeat it”, the vast amount of knowledge acquired with CAR-T cells produced with viral systems will have to be remembered so that we are not condemned to experience again the past issues and, instead, to forthrightly improve the efficacy, safety and availability of gene edited CAR-T cells.

## Author Contributions

RS created the first concept of the manuscript, created the figure and tables, organized and distributed the headings and wrote the introduction and headings 1, 2, 6, 8 and 5, and revised the final manuscript. DW wrote headings 4, 5, 7 and 8, completed table 2, and revised the final manuscript. MC wrote headings 1 and 6. CS, wrote heading 7. UK wrote headings 2 and 3, and revised the final manuscript. All authors agree to be accountable for the content of the work. All authors contributed to the article and approved the submitted version.

## Funding

RS’s laboratory is financed by grants of the German Center for Infections Research (DZIF-TTU07.912 to R.S.), by the Cancer Research Center Cologne Essen (CCCE), by the German Cancer Aid (Deutsche Krebshilfe Nr. 70114234 to RS) and by the Jackson Laboratory (USA, grant LV-HLA IO). DW has received funding from the European Union’s Horizon 2020 research and innovation programme under grant agreement No 825392 (ReSHAPE-h2020.eu) and is generously supported by the German Federal Ministry of Education and Research (BIH Center for Regenerative Therapies), a Berlin Institute of Health (BIH) Crossfield project fund of the BIH Research Focus Regenerative Medicine and the SPARK-BIH program.

## Conflict of Interest

RS has filed a patent application for generation of CAR-T cells targeting lytic herpes infections and is a founding shareholder and scientific consultant of BioSyngen/Zelltechs Lpt Ltd. DW has filed multiple patent applications on CRISPR-Cas gene editing and adoptive T cell therapy. CS is consultant for Bristol Myers Squibb, Janssen and Novartis regarding CAR-T cell therapy and is participating in clinical CAR-T studies from Bristol Myers Squibb, Janssen, Novartis and Miltenyi Biotec and is cooperating with Miltenyi Biotec in the production of CAR-T cells. UK states that she is a consultant in immuno-oncology for AstraZeneca, Affimed, Glycostem, GammaDelta and Zelluna, and that she has collaborations with Novartis and Miltenyi Biotec regarding the production of CAR-T cells.

MC is co-inventor in granted and filed patents describing CAR-T cells with additional functions to counteract the tumor microenvironment.

## Publisher’s Note

All claims expressed in this article are solely those of the authors and do not necessarily represent those of their affiliated organizations, or those of the publisher, the editors and the reviewers. Any product that may be evaluated in this article, or claim that may be made by its manufacturer, is not guaranteed or endorsed by the publisher.
